# Predicting Product Preferences on Retailers’ Web Shops through Measurement of Gaze and Pupil Size Dynamics

**DOI:** 10.5334/joc.240

**Published:** 2022-10-04

**Authors:** Guus van Loon, Felix Hermsen, Marnix Naber

**Affiliations:** 1 Bloakes Intuitive Marketing Research, Steenweg 54, 4181AM Waardenburg, The Netherlands; 2 Neurolytics, Europalaan 400-4, 3526KS, Utrecht, The Netherlands; 3 Experimental Psychology, Helmholtz Institute, Faculty of Social and Behavioral Sciences, Utrecht University, Room H0.25, Heidelberglaan 1, 3584CS Utrecht, The Netherlands

**Keywords:** Pupillometry, pupil dilation, decision-making, product preferences, gaze

## Abstract

Previous studies used gaze behavior to predict product preference in value-based decision-making, based on gaze angle variables such as dwell time, fixation duration and the first fixated product. While the application for online retail seems obvious, research with realistic web shop stimuli has been lacking so far. Here, we studied the decision process for 60 Dutch web shops of a variety of retailers, by measuring eye movements and pupil size during the viewing of web shop images. The outcomes of an ordinal linear regression model showed that a combination of gaze angle variables accurately predicted product choice, with the total dwell time being the most predictive gaze dynamic. Although pupillometric analysis showed a positive relationship between pupil dilation and product preference, adding pupil size to the model only slightly improved the prediction accuracy. The current study holds the potential to substantially improve retargeting mechanisms in online marketing based on consumers’ gaze information. Also, gaze-based product preference proves to be a valuable metric in pre-testing product introductions for market research and prevent product launches from failure.

## Introduction

Websites typically track mouse behavior of visitors as a measure of the distribution of an observer’s attention. This information can be used to investigate whether products, banners, or other regions of interest receive the expected attention. As webcam-based eye-tracking improves due to camera developments such as the implementation of hyperspectral (infrared) and high resolution chips, and improved gaze estimations (Asteriadis, Karpouzis, & Kollias, 2013; Cristina & Camilleri, 2018), it is not unthinkable that eye-tracking becomes a mass application in online market research. As gaze directly probes a person’s attention (Weichselgartner & Sperling, 1987), it may serve as a useful alternative to mouse-tracking (Johnson, Mulder, Sijbinga, & Hulsebos, 2012; Katerina, Nicolaos, & Charalampos, 2014). Here we investigate whether eye-tracking can indeed be of use to specifically online web shops.

Many studies have examined people’s gaze behavior when interacting with consumer goods. Distinct eye gaze patterns emerge depending on the amount of information, several design factors and the presence of a shopping goal. For instance, when interacting with one product without having an explicit goal, two distinct gaze patterns (i.e., spatial paths of sequential fixations on distinct stimulus locations) were distinguished in the first 1.5 second after the interaction. The first and most dominant gaze pattern is based on visual saliency. Visual saliency is the ability of an item on a screen or in the real environment to stand out from its surrounding based on low level visual features and attract more fixations correspondingly (Itti & Koch, 2001). So, it is primary the surface size and color of packaging elements that attracts immediate attention (Piqueras-Fiszman, Velasco, Salgado-Montejo, & Spence, 2013; Rebollar, Lidón, Martín, & Puebla, 2015). And gaze is more often attracted away from relevant items, when the surrounding holds more detailed information (B. Zhang & Seo, 2015).The secondary gaze pattern dependents on the viewers writing system, e.g. top left to bottom right in Western countries (Rebollar et al., 2015).

When presented with multiple products at once, which product is visited most depends on factors like product size and shape, packaging colors, the brand logo (Husić-Mehmedović, Omeragić, Batagelj, & Kolar, 2017). The latter two are especially of importance when size and shape are comparable across products (Atalay, Bodur, & Rasolofoarison, 2012). Other factors that affect attention are the product’s location relative to other products (Atalay et al., 2012), horizontal or vertical orientation of the products (Deng, Kahn, Unnava, & Lee, 2016), in-store shelf placement (Chen, Burke, Hui, & Leykin, 2021) and the number of shown products (Vu, Tu, & Duerrschmid, 2016).

In addition, visual saliency does (indirectly) affect the outcomes of consumer decisions (Gere et al., 2020; Lohse, 1997; Navalpakkam, Kumar, Li, & Sivakumar, 2012). A decision is reached quicker when the preferred options are the most salient (Nyamsuren & Taatgen, 2013) because bottom up attention excludes non-salient items from the consideration set (Wastlund, Shams, & Otterbring, 2018). The bias to choose for a visual salient item is stronger when making decisions under time pressure, while cognitive load is high or when choosing between low preferred items (Milosavljevic, Navalpakkam, Koch, & Rangel, 2012).

Although stimulus properties play an important role in the control of visual attention via bottom up processes (Itti & Koch, 2001; Nothdurft, 2000), gaze behavior is mostly dependent on goal directed attention (Bialkova & van Trijp, 2011; M. Hayhoe, 2000; M. M. Hayhoe, Shrivastava, Mruczek, & Pelz, 2003; Land, Mennie, & Rusted, 1999; Wolfe, 1994). Within the context of food images and advertising, changing the goal of a specific task affects the viewing duration, the number of fixations and the location of the first fixation (Rayner, Miller, & Rotello, 2008; Vu et al., 2016). Likewise, changing the complexity of the task goal affects the number of products that are seen in subsequent in-store decisions (Wästlund, Otterbring, Gustafsson, & Shams, 2015).

Beyond product designs, specific gaze behavior has also been linked to value-based decision-making, particularly in the context of consumer choices. While the first fixation on a set of choices has no direct influence on a decision (Meißner, Musalem, & Huber, 2016; van der Laan, Hooge, de Ridder, Viergever, & Smeets, 2015), the eventual chosen item is looked at longer and more often. This phenomenon, called ‘gaze bias’, leads to a reinforcement of item preference when getting closer in time to the decision moment (Shi, Wedel, & Pieters, 2013; Shimojo, Simion, Shimojo, & Scheier, 2003).

Gaze bias was found in laboratory task paradigms (Atalay et al., 2012; Cavanagh, Wiecki, Kochar, & Frank, 2014; Chandon, Hutchinson, Bradlow, & Young, 2009; Graham & Jeffery, 2012; Jantathai, Danner, Joechl, & Dürrschmid, 2013; Russo & Leclerc, 1994; Schotter, Berry, McKenzie, & Rayner, 2010), over time in relation to newspapers advertisements (J. Zhang, Wedel, & Pieters, 2009), as in real-world supermarket settings (Gidlof, Anikin, Lingonblad, & Wallin, 2017). In the latter, increased viewing time and frequency was directly related to buying a product. Accordingly, manipulating the gaze duration for a choice option increases the likelihood of being chosen (Armel, Beaumel, & Rangel, 2008; Shimojo et al., 2003). In line with gaze bias for preferred items, increased attention for aversive items decreases the chance of being chosen (Armel et al., 2008; Schotter et al., 2010).

Gaze patterns have been used to model consumer choice in highly experimental settings. The total fixation duration (Krajbich, Armel, & Rangel, 2010; Krajbich & Rangel, 2011; van der Laan et al., 2015) and first fixation duration increase the probability of choice for an item. Also, the last fixation is a predictor of the ultimate choice, however only when this last fixated item is considered to be attractive. Looking at a choice item for half a second more increases the chance on being chosen with 25% (Krajbich et al., 2010; Krajbich & Rangel, 2011).

Gaze patterns have also been examined to predict subsequent actions in real life settings (Gere et al., 2016; Gidlof et al., 2017; Huang, Andrist, Sauppe, & Mutlu, 2015). Gidlof and colleagues (2017) were able predict the actual purchase in a supermarket based on the total dwell time for a product. Total dwell time is here referred to as *the total time gaze is staying on a product*, and may include several fixations. Noteworthy, adding additional variables to the model, such as the number of product facings, product saliency, vertical shelf position and the product’s popularity and quality, did not improve the prediction.

Another study investigated multiple gaze behaviors to predict ingredient preference in sandwich shop decision-making simulation (Huang et al., 2015). In order to achieve highest predictability, the following gaze behaviors were examined: total duration of all fixations toward the ingredient; most recent fixation towards the ingredient; number of fixations; first fixation duration toward an ingredient. Again, looking longer towards an item was strongly related to the ultimate choice. Interestingly, the authors were able to predict decisions in real time, on average 1.8 seconds before a choice was expressed by a participant.

These applied studies investigated food preferences. For the sake of generalization, we here aim to study product preferences in a web shop environment. Furthermore, we aim to extend the set of potential predictive gaze behaviors with pupil size. Previous literature suggests that the pupil dilates when people detect a target or indicate to choose for one of several options (Einhauser, Koch, & Carter, 2010; Naber, Stoll, Einhauser, & Carter, 2013; Privitera, Renninger, Carney, Klein, & Aguilar, 2010; Strauch, Hirzle, Van der Stigchel, & Bulling, 2021). It is generally accepted that pupil dilation is a result of noradrenergic locus coeruleus activity (Breton-Provencher & Sur, 2019; Joshi, Li, Kalwani, & Gold, 2016; Liu, Rodenkirch, Moskowitz, Schriver, & Wang, 2017; Murphy, O’Connell, O’Sullivan, Robertson, & Balsters, 2014; Reimer et al., 2016). Fluctuations in pupil size can be an indication of different mental processes (for a review, see: Binda & Murray, 2015; Joshi & Gold, 2020; Mathot, 2018; Strauch, Wang, Einhäuser, Van der Stigchel, & Naber, 2022), such as memory load (Kahneman, 1966), cognitive effort (Alós-Ferrer, Jaudas, & Ritschel, 2021), conflict processing (van Steenbergen & Band, 2013), surprise (Preuschoff, t Hart, & Einhauser, 2011), and (emotional) arousal (Reimer et al., 2014; Reuten, van Dam, & Naber, 2018; Vinck, Batista-Brito, Knoblich, & Cardin, 2015). All are related to sympathetic nervous system activity (Bradley, Miccoli, Escrig, & Lang, 2008). The role of pupil dilation in value-based decision-making has been a neglected factor in marketing studies to date, marking it as an uninvestigated tool in an applied setting.

In summary, gaze behavior *and* potentially also pupil size hold valuable information to “read” the choice process and predict decisions, both in laboratory and real-life settings. However, gaze and pupillometry have neither been investigated in combination, nor in the context of increasingly popular online retail shops, such as Amazon, Ebay, AliExpress and similar web shops. This is why we here aim to investigate the role of dwell times and pupil dilation in consumer choice in the context of online retail shops. Based on above mentioned literature, we hypothesize that *dwell* and *pupil size* properties will be predictors of product preference.

## Methods

### Participants

We invited a total of sixty-three individuals (age: M = 22.8, SD = 3.0, range: 18–23; 31 women) to participate in the experiment. Participants received study credits for participation. All participants had normal or corrected to normal vision, gave their informed written consent before participation, were naive to the purpose of the experiment, and were debriefed about the purpose afterwards. The current study is compliant with the ethical principles of the Declaration of Helsinki and was approved by the faculty ethical review board of the University of Utrecht. The experiment lasted approximately one hour.

### Apparatus, Setup and Instructions

Stimuli were produced by a MSI GL62 laptop computer (Micro-Star International, Zhonghe, New Taipei, Taiwan), operating on Windows 7 (Microsoft, Redmond, WA, USA), with Tobii Pro Studio software version 3.4.8 (Tobii AB, Karlsrovagen, Danderyd, Sweden). The desktop computer screen had a resolution of 1920 × 1080 pixels, diagonal screen size of 15.6 inches (width: 13.6 inches, height: 7.7 inches) and a refresh rate of 60 Hz. The eye tracker was a Tobii X2-30 Compact with firmware version 1.2.2, enabling gaze and pupil tracking at a 30Hz rate. Participants were sitting in a well-lit room (standard office lights from the ceiling) on a non-moving chair behind an office desk. They were facing a laptop on an approximate distance of 25 inches from the eye tracker, in a comfortable position to read the text on the screen and reach the mouse and the keyboard. Head movement of the participants was not restrained. Distance to the eye tracker was verified with the Track Status window of the Tobii Pro Studio software before starting a recording. Participants were instructed to remain quiet and sit up straight throughout the whole experiment, without moving their head away from the screen or placing their hands in front of their faces.

### Stimuli, Experimental Design, and Procedure

**Figure 1:**
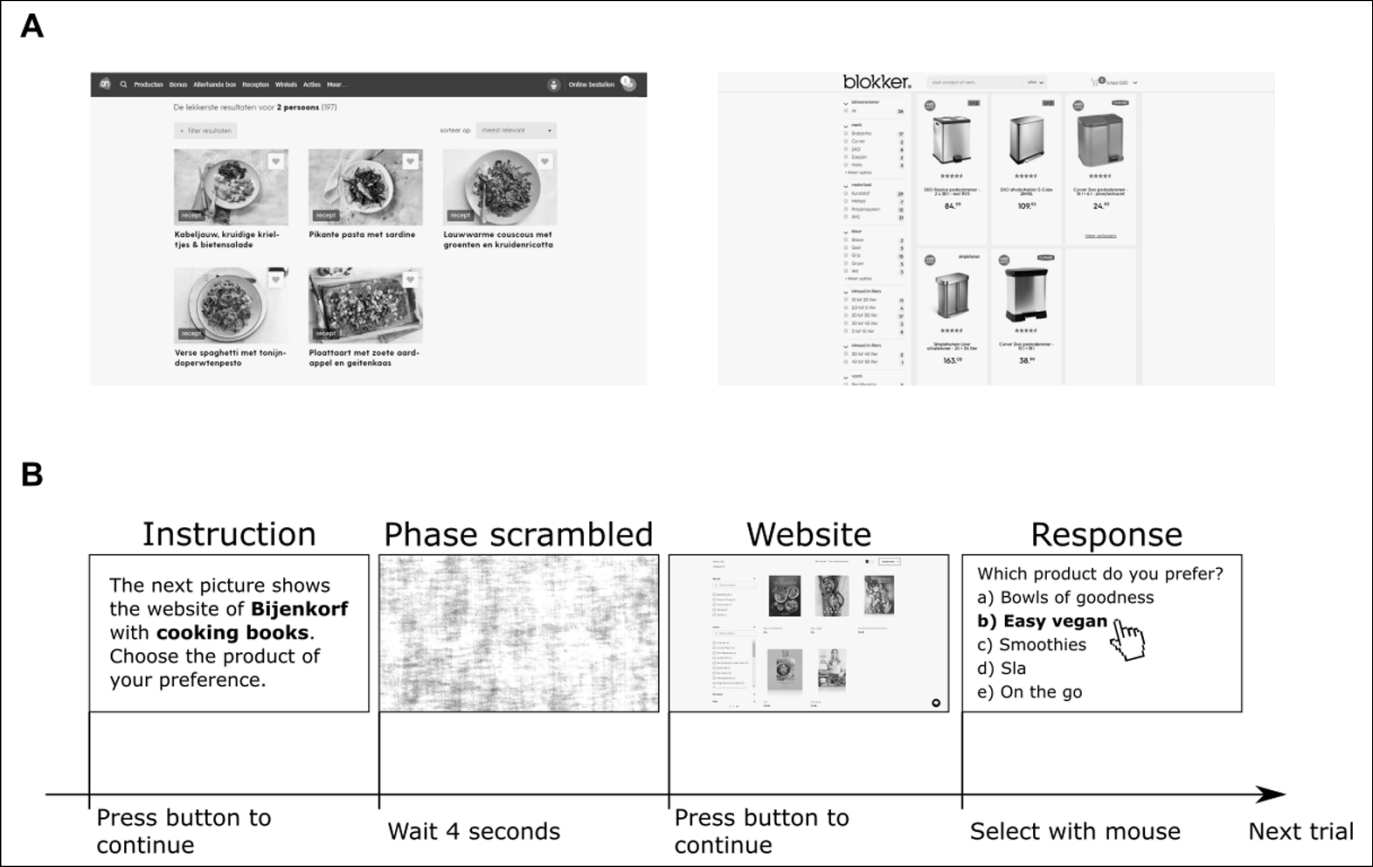
**Stimuli and procedure. (A)** Two examples of pictures shown to the participants. All pictures were equal in luminance. **(B)** Example procedure of a single trial. A trial started with the presentation of a short instruction, a phase-scrambled version of the web shop, the actual web shop, and then the multiple-choice question about which product is preferred by the participant.

The experiment consisted of the presentation of 60 pictures of web shops (for examples, see Figure 1A; for all pictures, see **Supplementary Figure S1**). The pictures were collected online and were based on Dutch versions of web shops of a variety of retailers. We modified the content of the web shops to ensure that each web shop displayed five products and few words (M = 27.6, SD = 14.9, range = 5 – 95 words) explaining product properties to prevent distorting effects of product and word number. All pictures had a white background and were equalized in luminance to prevent distorting effects on pupil size. Luminance equalization was achieved by subtracting the median of all pixel values per image and adding the mean of all median pixel value across all images (Mean = 225/255, Median = 247/255, SD = 0.22 in 8-bit values). The gamma of the screen was set to 2.2 and reached a maximum luminance of 230 cd/m^2^.

Each eye tracking recording started with a check if the eye tracker picked up the signal of both eyes, using the Track Status window in Tobii Pro Studio. A standard calibration procedure was conducted to improve the accuracy and precision of the eye tracker signals (Holmqvist et al., 2022). The calibration consisted of an animated red circle with a black dot in the middle that moved over a grey screen. The circle stopped at five distinct spatial locations across the screen (four in each corner, one in the middle) and changed in size from large to small during movements between each location. Calibration data was collected while the circle was at a fixed position and at its minimal size. Participants were instructed to follow the black dot in the red circle as accurately as possible, without making blinks or moving the head. When the calibration did not meet the desired quality (accuracy), the eye tracker was recalibrated.

Before the presentation of each picture, we presented a phase-scrambled version of each web shop’s picture (Figure 1B) for 4 seconds to stabilize spontaneous fluctuations in pupil size and prevent trial-to-trial crossover effects. Phase scrambling was accomplished by (i) transforming the picture to frequency domain with a 2D Fourier transform resulting in an amplitude and phase matrix, (ii) adding a random phase to the phase matrix, and (iii) converting the matrices back to normal with an inverted Fourier transform resulting in a 2D phase-scrambled picture.

After the presentation of each web shop (for a visualization of the procedures, see Figure 1B), a multiple-choice question “Which product do you prefer?” with five answer options (randomized order per trial). The answer to this question was used to investigate whether we could predict product preferences based on gaze and pupil size.

### Analysis

Two participants were excluded from the study because they looked at the images to shortly (total experiment time less than 30 minutes). The analysis started with the manual selection of product areas (i.e., sample-based areas of interest) by author GVL. Product areas for all five products of each web shop image were determined by selecting areas through the serial selection of the most top left pixel to the most bottom right pixel. An area included the text below the product images, i.e. titles, product information and pricing (Figure 1A). Areas were highly similar in size and no space was left between product areas, meaning that areas of horizontally aligned products had similar y-coordinates and areas of vertically aligned products had similar x-coordinates. The total product area included the product areas of all five products. The sample-based eye position on the screen was mapped on both the single product areas and total product area per web page.

The eye tracking signal was recorded with an average data loss across participants of 7.6% (SD = 4.4%, range: 2% – 20%). From the continuous eye position and pupil size recordings, we calculated the gaze angle variables: (1) total dwell time (i.e., the sum of all individual dwells, with a dwell being defined as the duration of someone looking at a product area, including multiple fixations, blinks and saccades), (2) median dwell duration (the median of the total dwell time across participants), (3) number of dwells; and the pupil variables: (1) mean pupil size (pupil size is equal to the pupil diameter as outputted by the eye tracker), (2) mean pupil size relative to the phase scrambled baseline, and (3) mean detrended pupil size per product per web shop image. A dwell was determined as each time period that gaze was maintained uninterruptedly on a single product area. We interpolated blink periods with MATLAB’s pchip method. For the latter pupil variable, pupil size was detrended to remove the typically gradual, slow (over a period of multiple minutes) decrease in pupil size during an experiment. By removing the trend, baseline pupil size cannot contain confounds of experiment time. Detrending consisted of subtracting a low-pass Butterworth filtered signal (cut off frequency: 0.1Hz; 1st order) from the original pupil size signal. The potential effect of foreshortening error, due to narrowing of the pupil as a function of the deviation in angle between the eye’s and camera’s orientation, was not controlled for but the locations of preferred versus nonpreferred items varied across stimuli and individuals to a degree that prevented biases. Rank scores were based on value ranking, ordering products from 1 (highest) to 5 (lowest) per gaze/pupil variable. For example, a product with the longest dwell time would receive rank 1. Next, the percentage chosen versus not chosen product was computed per rank and per gaze/pupil variable.

## Results

**Figure 2:**
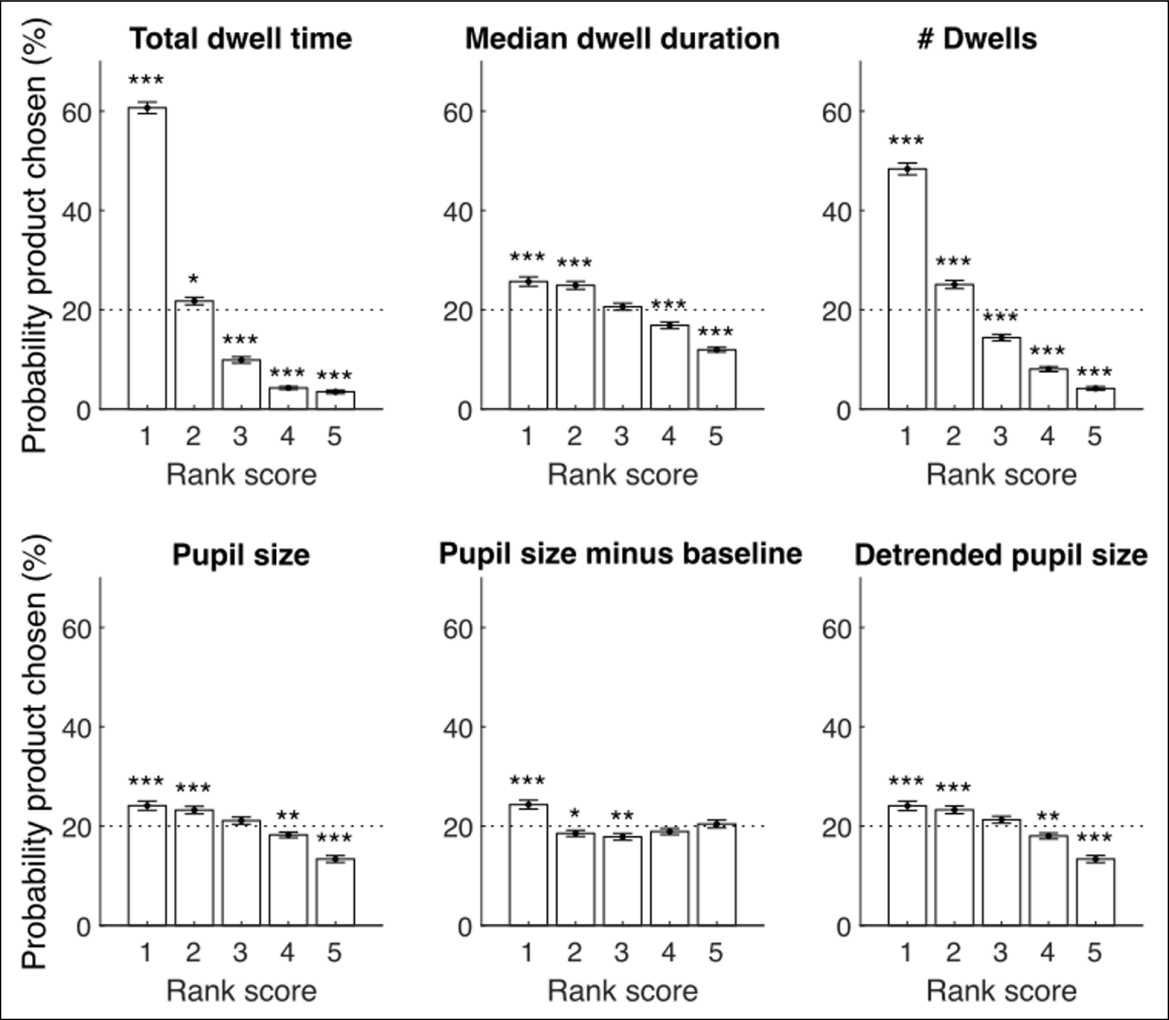
**Rank score results.** Percent of trials (y-axis) per rank score (x-axis) per independent variable (panel). A rank score of 1 means that the product was dwelled on most and longest, and evoked largest pupil size during viewing, while a rank score of 5 means it was dwelled on less often, shortest, and evoked smallest pupil size during viewing.

The independent variables were evaluated on how well they could predict whether a product was chosen or not (Table 1). First, we compared total dwell time, median dwell duration, and dwell frequency for preferred versus nonpreferred products across participants. These comparisons showed that all these gaze angle variables significantly differed between preferred and nonpreferred products. The participants dwelled on the preferred product more often (~10 dwells per web shop image) and longer (~0.5s per dwell), resulting in longer total dwell time (~4s) as compared to nonpreferred products (~7 dwells, ~0.4s per dwell, ~2s total dwell time). When participants viewed a preferred product, the pupil also increased in size slightly more than when they viewed a nonpreferred product (0.02mm; 1% increase; for pupil responses to view onsets of preferred versus nonpreferred products, see **Supplementary Figure S2**). As also shown in Table 1, the AUC of the ROC, calculated to describe the degree overlap between value distributions of preferred versus nonpreferred products of each independent variable (for details, see Methods – analysis), indicated that the gaze angle variables, and especially the total dwell time dissociated best between a preferred and nonpreferred product. The product that scored highest on each of the independent variables was also the preferred product in a large proportion of trials (Figure 2). In sum, pupil size and especially gaze angle variables showed potential in predicting which product would be chosen as the preferred product.

**Table 1:** Means (M), standard deviation (SD), outcomes of paired t-tests (t, p), and effect sizes (AUC) of comparisons between preferred and nonpreferred products per independent variable.


INDEPENDENT VARIABLE	M (SD) PREFERRED	M (SD) NONPREFERRED	*t* (DF)	*p*	AUC

Total dwell time	3.937 (1.454)	2.221 (0.845)	19.16 (60)	<.001	0.75

Median dwell duration	0.509 (0.16)	0.429 (0.123)	7.68 (60)	<.001	0.53

Number of dwells	10.302 (4.915)	6.546 (3.042)	14.08 (60)	<.001	0.69

Mean pupil size	3.260 (0.455)	3.242 (0.454)	7.4 (60)	<.001	0.54

Mean pupil size minus baseline	–0.008 (0.051)	–0.015 (0.038)	1.91 (60)	.061	0.52

Mean detrended pupil size	3.399 (0.455)	3.381 (0.453)	7.45 (60)	<.001	0.54


Next, we aimed to investigate whether the gaze angle variables *combined* successfully predicted the preferred product per web shop better than the best gaze angle variable *individually*. An ordinal linear regression model was computed per participant, with either only dwell time, all gaze angle variables, or all gaze and pupil variables as predictors of the preferred product. We reported the betas and accuracies of the models, averaged across all participants, in Table 2. We reported the comparison of accuracies across the models (i.e., AUC) in Table 3. The difference in accuracy between the “total dwell time” model and “all gaze angle” model was 0.06 in AUC, meaning an 8 percent increase in prediction accuracy for the “all gaze angle variables” model. In addition, the model that combines gaze angle and pupil size variables as predictors had higher prediction accuracies than the “all gaze angle” model (difference: 0.01 AUC; 1% increase). In conclusion, a combination of all gaze angle variables as predictors improves the accuracy in determining which product was chosen by participants. The addition of pupil size measurements to the set of predictors only slightly improved the prediction accuracy.

**Table 2:** Mean (M) and standard deviation (SD) of regression weights (betas, estimates) across participants of the probability to prefer a product per model that either uses all gaze angle variables or all gaze angle and pupil variables combined as predictors.


MODEL	AUC (M ± SD)	INTERCEPT	VARIABLE	BETAS (M ± SD)

All gaze angle variables	0.81 ± 0.05	0.03 (0.05)	Total dwell time	–1.19 (0.52)

			Median dwell duration	–0.63 (0.41)

			Number of dwells	–1.23 (0.56)

All gaze and pupil variables	0.82 ± 0.05	0.92 (0.40)	Total dwell time	–1.19 (0.57)

			Median dwell duration	–0.68 (0.46)

			Number of dwells	–1.31 (0.63)

			Mean pupil size	1.56 (8.40)

			Mean pupil size minus baseline	–0.07 (0.33)

			Mean detrended pupil size	–1.69 (8.45)


**Table 3:** Comparison of prediction accuracies across models described in Table 2.


MODEL 1	MODEL 2	DIFFERENCE AUC (M± SD)	*t* (DF)	*p*

Total dwell time	All gaze angle variables	0.06 (0.04)	14.17 (60)	<0.001

All gaze angle variables	All gaze and pupil variables	0.01 (0.01)	7.86 (60)	<0.001


## Discussion

In the present study, we predicted consumer choice on retail web shops by measuring gaze angle and pupil size variables. We first found that gaze angle variables were predictive of subsequent product choice. As expected, people look at preferred products longer and more often. A novel finding in this research is that pupil size was also predictive for product choice. Although weaker than gaze variables, it did slightly improve the accuracy of the prediction. A relevant element of our study is that the participants made choices between products presented on existing retail web shops. Variations in the amount of visual information and products across the web shops were taken into account as well. This ensured real-life like decision-making and high ecological test validity. To our knowledge, no previous study predicted product preference based on gaze behavior on retail web shops. Hence, the present study is a novel contribution to the field of consumer decision-making.

The findings in this study are in line with previous literature suggesting that the eventual chosen option is attended to the most during the decision process (Orquin & Mueller Loose, 2013; Russo & Leclerc, 1994; Shimojo et al., 2003; van der Laan et al., 2015). Three previous studies used this approach to predict product preference, all in a food preference context (Gere et al., 2016; Gidlof et al., 2017; Huang et al., 2015). Gere and colleagues (2016) applied a multi-alternative forced choice paradigm (4AFC) with four food items of the same category as the choice options. A typical laboratory task presenting well-structured objects to the participants on a plain background. The study is particularly of interest since it evaluated six gaze parameters, selected the three most promising and compared the outcomes of thirteen applied prediction models. This approach resulted in a prediction with an extremely high accuracy of 99.75% for the k-Nearest Neighbor (KNN) model. The current study also studied other models in preliminary analyses (data not shown), including the KNN model, but these achieved similar results as the here reported linear regression model. Although the study by Gere et al. (2016) is well thought out and the outcomes are outstanding, they should be evaluated in the context of a highly controlled task-paradigm, with relatively simple images and without real-life distractions from in-store materials or website elements. Also, it is unclear how overfitting was prevented in the KNN model. Moreover, the other, above-mentioned choice prediction studies were carried out in a physical environment. In an in-store experiment, supermarket product purchase was predicted with 88% certainty (Gidlof et al., 2017). In a sandwich shop simulation study, in which participants had to pick preferred toppings, the chosen topping was accurately predicted in 76% of all choices (Huang et al., 2015). As such, it is not unlikely that the use of real-life stimuli and environments adds noise to the decision process and thus the predictability of consumer choices based on eye-tracking variables.

It’s notable that the most accurate outcomes were found in the studies that incorporated gaze parameters that capture more than merely fixations. In contrast to dwell times, measuring only fixations does not incorporate the time periods when people make saccades. It is assumed that perception is suppressed and impaired during saccades (Bremmer, Kubischik, Hoffmann, & Krekelberg, 2009; Thiele, Henning, Kubischik, & Hoffmann, 2002), suggesting that it is only sensible to calculate fixation durations and frequency rather than dwell time that includes the saccade periods as well (i.e., dwell time accumulates already when gaze enters the product area rather than when a saccade ends and a fixation starts). That dwell time includes higher predictive power might be due to the fact that some information about a saccadic end point (e.g. a preferred product) is already processed before and during a saccade is performed. This pre-saccadic information acquisition is likely the result of pre-saccadic shifts of attention (Godijn & Theeuwes, 2003; Mathot & Theeuwes, 2010; Rolfs, Jonikaitis, Deubel, & Cavanagh, 2011), a process believed to facilitate a smooth perception of the world, which explains why our perception is experienced as coherent across time rather than physically abrupt as is the case at the level of the retina during eye-movements. When people are making many saccades on or around a preferred product, the total fixation time may be short but the total dwell time would still be long. This was also the reason why we focused on dwell time rather than fixations. Although out of scope of the current study, future studies may also implement sophisticated gaze pattern analyses to improve predictions.

That pupil size is indicative of product preference during consumer decision-making was never explicitly reported in the literature before. However, several strong indications exist. When multiple stimuli are presented serially, the pupil dilates the moment people decide to choose (Einhauser et al., 2010; Naber et al., 2013). Human performance on Go/NoGO tasks showed larger pupil size on Go-trials, and for ‘Yes’ over ‘No’ trials, especially when stimuli were relevant to the task-goal (de Gee et al., 2017; de Gee, Knapen, & Donner, 2014; Strauch, Greiter, & Huckauf, 2018; Strauch, Koniakowsky, & Huckauf, 2020). In rodents, pupil dilation predicted choice with 80% certainty (Lee & Margolis, 2016). Moreover, two studies that examined the relationship between pupil size and website behavior, showed that pupil dilation was predictive of clicking, over non-clicking, on an object on a website. The first study reached 82% accuracy when applying an artificial neural network model (Jadue, Slanzi, Salas, & Velasquez, 2015), while a follow-up study performed best, in terms of 71% accuracy, with a regression model (Slanzi, Balazs, & Velásquez, 2017).

An explanation why pupil size acts as a predictor of a subsequent product choice could be found in the response facilitation theory, stating that increased arousal facilitates the formation of a behavioral reaction (Allen, Kenrick, Linder, & McCall, 1989). Consistently, arousal in the neocortex facilitates the behavioral response during decision-making (Clayton, Rajkowski, Cohen, & Aston-Jones, 2004; Nieuwenhuis, Aston-Jones, & Cohen, 2005) and is particularly relevant when choice variability is high or when choice-bias needs to be suppressed (de Gee et al., 2017; Murphy, Vandekerckhove, & Nieuwenhuis, 2014). Hence, this could mean that pupil-linked arousal fulfills an important role in the execution and control of the decision outcome.

Additionally, our findings tie in well with a previous study on wine brands, wherein product liking, brand liking and willingness to pay were positively related to pupil dilation (Ramsøy, Jacobsen, Friis-Olivarius, Bagdziunaite, & Skov, 2017). Interestingly, the authors also find increased arousal (and pupil dilation) for brands that are perceived with a negative valence, and attribute a major modulating role for body posture in the relationship between pupil size and preference. Increased pupil size independently of the valence is also seen when people view (emotionally) arousing stimuli (Bradley et al., 2008; Wang et al., 2018).

As proposed in a recent study, pupil size can reflect more than one cognitive process during a perceptual decision-making task. Strauch and colleagues (Strauch et al., 2021; Strauch et al., 2020) showed that pupil size first reflects relevance of a stimulus and thereafter a value judgement. Hypothetically, the prediction of consumer choices based on pupil dilation might involve multiple underlying processes too, including response facilitation and stimulus induced arousal. Future research should examine the potential steps that may exist in the decision process of product choice, and are linked to early versus late pupil dilation during this decision process. Knowing when the pupil dilates in relation to stimulus onset time and the moment of the decision may hold important information to improve future prediction models.

The goal of the current study was to predict product preference on retail web shops based on gaze behavior. One requirement in the achievement of this goal was that many kinds of different gaze behaviors could be captured, including pupil dilation. Because pupil size changes with changing brightness, all web shop images were equalized in luminance. A limitation of this approach is the loss of color, wherefore one could question how well the results would generalize to colorful web shops. Although out of scope of the current study, it would be valuable to replicate the study with full colored web shops.

Although presenting the web shop as images has several advantages in terms of control over the stimulus properties, and the absence of additional motor execution induced pupil changes (Richer & Beatty, 1985; Strauch et al., 2021), it also introduces two limitations. Most importantly, it does not completely match the experience of browsing through and interacting with a real web shop. And secondly, it was not possible to indicate a choice directly in the shop. Instead, participants answered to a question on the subsequent screen. A future study may include product search and purchase on actual websites to come even closer to natural behavior. Unless these limitations, the ecological test validity of the current research design is high compared to the alternative forced choice (AFC) tasks, a research paradigm often applied to investigate the consumer choice process. The AFC is a severely simplified representation of reality due to the limited number of choice options and the basic visual presentation of stimuli.

A last limitation comes with the relatively large number of choices as made by the participants. Although the attempt to include generally known and neutral retail web shops, it was highly likely that some of the 60 chosen products were not relevant to the participant.

The outcome of the current study is relevant for the rapidly growing online retail market, to get a better understanding of consumer decision-making on ecommerce websites. These findings can be translated into relevant applications for the fields of online marketing and market research. Predicting product preference by measuring gaze behavior (of course after approval) during online shopping could increase the relevance of the additional recommended products in a web shop, improving both the shopping experience of the customer and increasing sales for the retailer. Likewise, information about personal preferences acquired by measuring gaze behavior could make interest-based retargeting more efficient (Lambrecht & Tucker, 2013). Furthermore, gaze-based product preference predictions can become a valuable addition to current market research methods. It has the potential to be a trusted measurement in the pre-examination of new product introductions. And, more importantly, it may prevent product introductions from failure.

In conclusion, our results show that product choice on retailer web shops can be predicted with high accuracy based on gaze behavior. Of all tested gaze measurements, dwell time on a product is clearly the most predictive. A novel finding in our study is that pupil size is also predictive for consumer choice but only to a small degree. Pupil dilation may be interpreted as the increase of cortical arousal that facilitates the decision process. Taken together, this research provides relevant new insights about product preference predictions applied in a real-world retail web shop case.

## Data Accessibility Statement

Research data is available on DOI: 10.17605/OSF.IO/8XQZR. Summary: This project entails the testing of 63 participants that looked at 60 snapshots of a variety of Dutch retailer websites displaying a variety of products. Participants had to select the product they liked most. An eye-tracker recorded their gaze and pupil size.

## Additional File

The additional file for this article can be found as follows:

10.5334/joc.240.s1 Appendix. Supplementary Figures.
