# Supplementary material for: Predicting Product Preferences on Retailers’ Web Shops through Measurement of Gaze and Pupil Size Dynamics
Source: J Cogn. 2022 Oct 4;5(1):45. doi: 10.5334/joc.240 (PMC9541120; doi:10.5334/joc.240)
Supplement: Appendix. — Supplementary Figures. [file joc-5-1-240-s1.pdf]

Appendix: Supplementary Figures

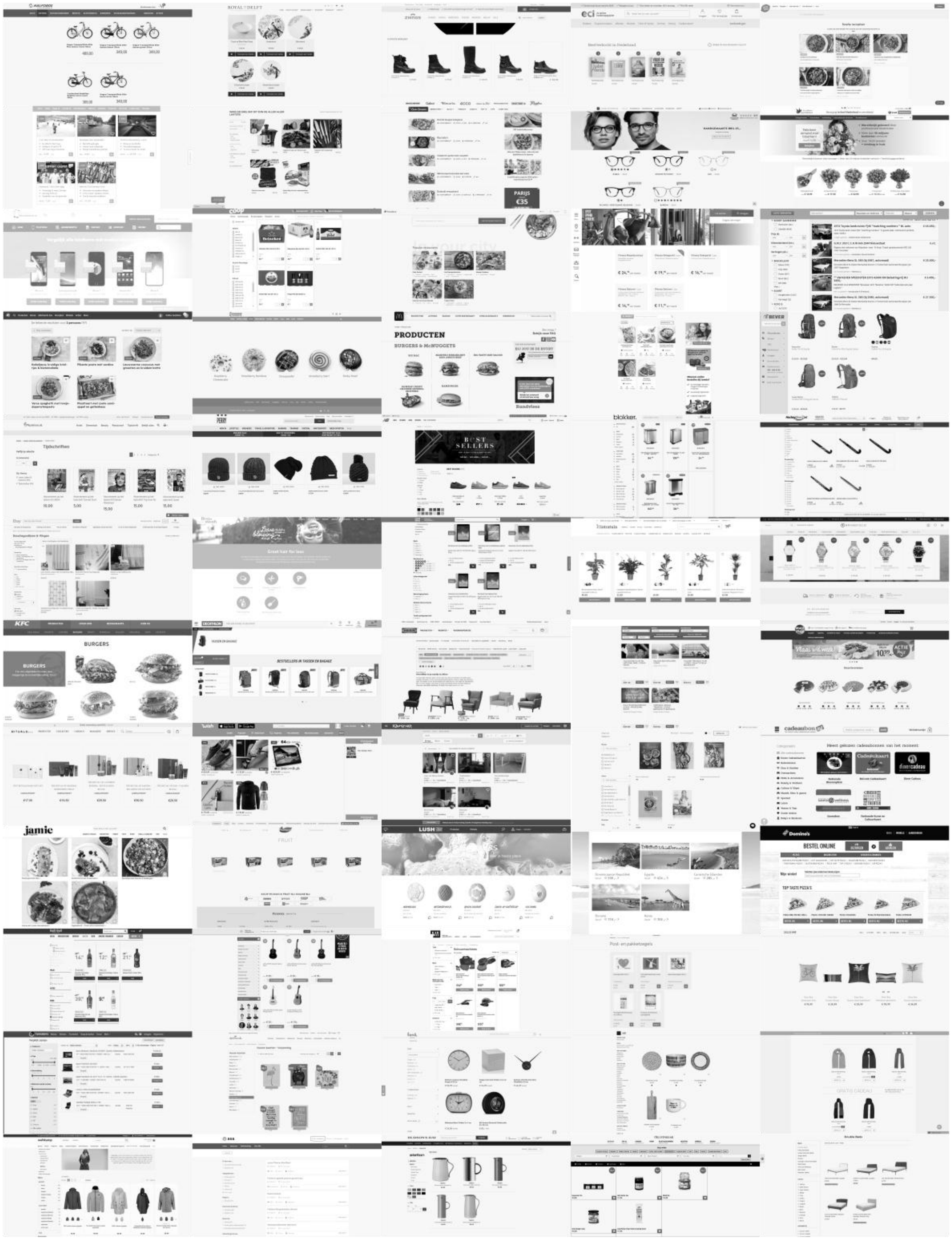

Supplementary Figure S1. *Tiled view of all web shop pictures presented during the experiment.*

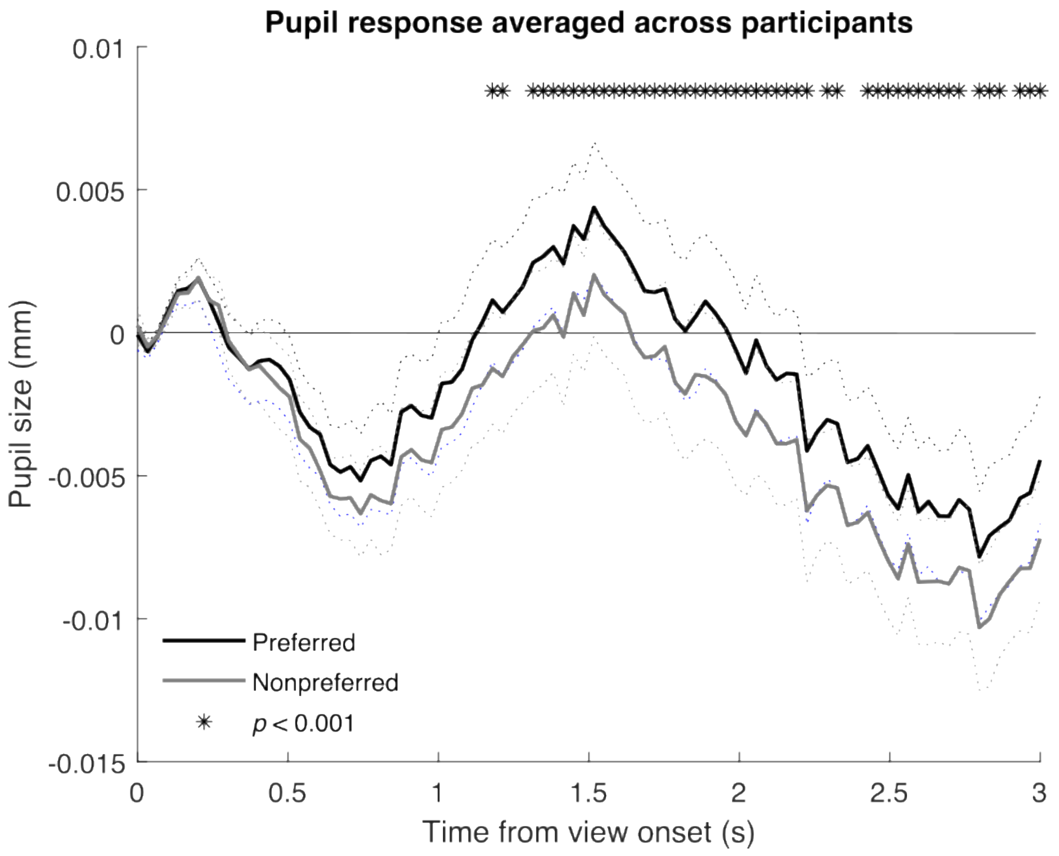

Supplementary Figure S2. Pupil responses during the viewing of preferred (blue) and nonpreferred products (red). Relative pupil size (y-axis) changed as a function of time from view onset (x-axis) of preferred or nonpreferred products. Relative means that the raw pupil size traces were subtracted by the average pupil size in the first 100ms of every view onset. The asterisks (magenta) indicate time points at which pupil responses for preferred products differed significantly from nonpreferred products (paired, two-tailed t-tests).
